# Emotion Control Predicts Internalizing and Externalizing Behavior Problems in Boys With and Without an Autism Spectrum Disorder

**DOI:** 10.1007/s10803-018-3519-8

**Published:** 2018-03-06

**Authors:** Marieke G. N. Bos, Sofia Diamantopoulou, Lex Stockmann, Sander Begeer, Carolien Rieffe

**Affiliations:** 1 Department of Developmental and Educational Psychology, Institute of Psychology, Leiden University, Wassenaarseweg 52, 2333 AK Leiden, The Netherlands; 2 Rivierduinen, Centre for Autism, Leiden, The Netherlands; 3 Faculty of Behavioural and Movement Sciences, Vrije Universiteit, Amsterdam, The Netherlands; 4 Foundation for the Deaf and Hard of Hearing Child, Amsterdam, The Netherlands; 5 Department of Psychology and Human Development, Institute of Education, University College London, London, UK

**Keywords:** Autism Spectrum Disorders (ASD), Longitudinal study, Comorbid psychopathology, Emotion regulation, Emotional control

## Abstract

Children and adolescents with Autism Spectrum Disorder (ASD) often show comorbid emotional and behavior problems. The aim of this longitudinal study is to examine the relation between emotion control (i.e., negative emotionality, emotion awareness, and worry/rumination) and the development of internalizing and externalizing problems. Boys with and without ASD (N = 157; age 9–15) were followed over a period of 1.5 years (3 waves). We found that baseline levels of worry/rumination was a specific predictor of later externalizing problems for boys with ASD. Furthermore, the developmental trajectory of worry/rumination predicted the development of internalizing and externalizing problems in both groups. Our findings suggest that worry/rumination may constitute a transdiagnostic factor underlying both internalizing and externalizing problems in boys with and without ASD.

**Electronic supplementary material:**

The online version of this article (10.1007/s10803-018-3519-8) contains supplementary material, which is available to authorized users.

## Introduction

Autism spectrum disorders (ASD) are characterized by social and communication difficulties and the presence of repetitive behaviors and interests (DSM V, APA 2013). In addition to these core impairments, up to 70% of the children and adolescents with ASD show comorbid psychiatric problems, like social anxiety and oppositional defiant disorder (e.g., Simonoff et al. 2008). Recently, there is a growing interest in the role of emotion control as a possible underlying mechanism that may explain these internalizing and externalizing behavior problems (Mazefsky et al. 2013).

The ability to control emotions is essential to navigate through daily hazards. It allows one to keep an optimal level of arousal in order to secure both social and personal goals (Chambers et al. 2009). Its development is affected by social experiences, and modeled through social learning. Indeed, from childhood onwards we learn how to control our emotions in a socially and culturally accepted way (Gullone et al. 2010; Morris et al. 2007; Southam-Gerow and Kendall 2002). However, children and adolescents with ASD have less access to the social learning environment and show deficits in the ability to control emotions (Mazefsky and White 2014; Rieffe et al. 2012; White et al. 2014). The aim of the current longitudinal study is to examine the role of three indices related to emotion control—negative emotionality, emotion awareness and worry/rumination—on the development of internalizing and externalizing behavior problems in children and adolescents with ASD, as compared to a typically developing (TD) control group.

Emotion control is an umbrella term that is used to describe several aspects related to the ability to down regulate emotional over-arousal in emotion-evoking situations. Problems in emotion control can be related to impairments in both emotion generation and in the process that relates to the ability to deal with emotions (Sheppes et al. 2015). Previous cross-sectional studies have shown that different indices of emotion control are related to internalizing and/or externalizing behavior problems. One index of problems in emotion control is the frequent experience of negative emotions, like fear, anxiety, and anger. This negative emotionality is a direct consequence of an inability to down regulate emotional over-arousal. Heightened levels of negative emotionality is associated with both internalizing (e.g., anxiety) and externalizing behavior problems (i.e., bullying and aggression) in TD youth and in children and adolescents with ASD (Pouw et al. 2013a; Rieffe et al. 2012; White et al. 2009, 2014). Another index of emotion control is emotion awareness. Emotion awareness relates to the ability to know how you feel and to link this feeling to an emotion-evoking situation is critical for the experience and regulation of emotions (Barrett et al. 2001; Rieffe et al. 2008). Indeed, not being able to differentiate between emotions and focusing too much on bodily symptoms of an emotional experience is related to more depressive symptoms, anxiety symptoms and somatic complaints in TD children and children with ASD (Rieffe and De Rooij 2012; Rieffe et al. 2008). In a similar vein, several studies indicate a relation between alexithymia and emotional problems in children and adolescents with ASD (for a review, see Bird and Cook 2013). Another index of emotion control that is related to the (in)ability to deal with emotional over-arousal is worry. Worry and rumination are highly related processes that are characterized by a chain of repetitive negative thinking, that increases the level of emotional over-arousal (Watkins 2008). The role of worry/rumination in youth with ASD is relatively understudied. This is remarkable since individuals with ASD have a propensity to perseveration and may therefore be uniquely susceptible to worry/rumination (Mazefsky et al. 2012; Patel et al. 2017). Worry/rumination are typically associated with the development and maintenance of internalizing behavior problems in TD youth (Nolen-Hoeksema et al. 2008). Likewise, we previously demonstrated a cross-sectional and longitudinal relation between worry/rumination and depressive symptoms in children and adolescents with ASD (Pouw et al. 2013b; Rieffe et al. 2014). Recently it has been shown that worry/rumination is also related to aggressive behavior in TD boys (McLaughlin et al. 2014). It is however unknown whether worry/rumination also contributes to disruptive behavior problems in children and adolescents with ASD.

Our knowledge of the role of emotion control on the development of internalizing and externalizing behavior problems in children and adolescents with ASD relies mainly on cross-sectional data. Even though these studies provide essential information for our understanding of this relationship, longitudinal studies are key to advance our knowledge on whether these relations hold over time. Therefore, we conducted a longitudinal study to test the relation between negative emotionality, emotion awareness, and worry/rumination with internalizing and externalizing behavior problems in 9–15 year old boys with and without ASD. We focused on this age range, given that social and emotional problems often increase during adolescence (Kuusikko et al. 2008; Paus et al. 2008).

We investigated three clusters related to internalizing problems: depression, anxiety, and somatic complaints, and one general cluster of externalizing problems: disruptive behavior. Participants and their parents filled in questionnaires about different aspects of emotion regulation and overall well-being at three time points (9 months in between each wave). Specifically, we aimed to test in both groups (1) whether emotion control contribute to the prediction of internalizing and externalizing behavior problems 18 months later, (2) examine the developmental trajectory of internalizing and externalizing behavior problems over time and (3) test the co-occurrence of the developmental trajectory of emotion control with the developmental trajectory of internalizing and externalizing behavior problems.

Based on the literature, we expect that negative emotionality, poor emotion awareness, and worry/rumination are related to more internalizing problems in both boys with and without ASD (Aldao et al. 2010; Barrett et al. 2001; Rieffe et al. 2008). For externalizing problems, we expect that negative emotionality and worry/rumination will have a positive predictive value for both groups (McLaughlin et al. 2014). Given the importance of emotion regulation for social functioning, the ability to employ effective emotion control is relevant for both groups. Nevertheless, the social impairments related to ASD might leave children and adolescents with ASD more susceptible to develop problems with controlling their emotions for which adequate socialization seems to be critical. Thus, the lack of social learning opportunities may reduce their capacities to control emotions and add to their vulnerability for developing social and emotional problem behaviors. Hence, we expect that the predictive relation between emotion control with internalizing and externalizing behavior problems is stronger for boys with ASD compared to their TD peers.

## Methods

### Participants

The current study was part of a larger project investigating the social-emotional development of typically developing children and children with less access to the social environment (children with hearing loss and children with ASD) (e.g., Broekhof et al. 2017; Netten et al. 2015; Pouw et al. 2013a, b; Rieffe et al. 2012, 2014). For the purpose of the current study, we used the data of TD boys and boys with ASD from whom parent-reports and self-reports were available at least at one time point.

The high functioning ASD sample included 66 children at T1. Inclusion criteria were: (i) ASD diagnosis on T1 according to the DSM-IV (APA 1994) based on the Autism Diagnostic Interview-Revised (Lord et al. 1994) by a child psychiatrist, (ii) IQ score above 80 and (iii) no additional DSM-IV diagnoses. Participants were recruited from specialized diagnostic and treatment center for children with autism in the Netherlands. A group of 89 TD children was recruited from primary and secondary schools in the Netherlands (see Table 1 for sample characteristics). Inclusion criteria for the control group was: (i) IQ above 80 and (ii) no DSM-IV diagnosis. All procedures were approved by the Ethical Committee of Leiden University and all parents provided written informed consent.

**Table 1:** Demographic characteristics of participants

	Total study population at T1 N = 156	
	ASD	Controls
No. of children	66	89
Subgroup ASD^a^		
Autism	20	
Asperger	7	
PDD-NOS	27	
MCDD	4	
Age		
Mean—in age (SD) at T1	11.65 (1.27)	11.39 (1.37)
Range—in years	9–15	9–15
Socioeconomic status (SD)		
Educational level^b^	3.84 (0.55)	3.62 (0.55)
Net income^c^	3.80 (1.59)	3.96 (1.43)
Nonverbal IQ^d^	11.59 (3.11)	11.01 (2.39)
IQ normscore picture arrangement^d^	11.45 (3.85)	11.11 (3.11)
IQ normscore block design^d^	11.73 (3.44)	10.92 (2.89)
Social responsiveness scale***	91.53 (26.95)	31.94 (19.62)

***p < .001

^a^Note, we did not have information on specific diagnosis for six participants in the ASD group

^b^1: no/primary education, 2: lower general secondary education, 3: higher general secondary education, 4: college/university

^c^1: < €15,000, 2: €15,000–€30,000, 3: €30,000–€45,000, 4: €45,000–€60,000, 5: > €60,000

^d^The presented IQ scores are age-corrected norm scores; the grand population mean is set to ten

### Measurements

#### IQ

Two nonverbal subtests (i.e., block design and picture arrangement) of the *Wechsler Intelligence Scale for Children-Third edition* (WISC III; Kort et al. 2002) were used to calculate a general measure of intelligence. The obtained scores were converted into age-corrected norm scores. The grand population mean is set to 10. The IQ subtests were not administered in 2 ASD and 5 TD boys due to time constraints.

### Predictors

#### Negative Emotionality

The mood list (Rieffe et al. 2004) is a self-report questionnaire that was used to assess children’s negative mood over the past 4 weeks. We used three subscales of the mood list: anger, fear and sadness. Each subscale consisted of 4 items on a 3-point rating scale (e.g., I never/sometimes/often feel angry). We used a total score for negative mood by taking the sum score of the negative items. Higher scores indicated dysregulated emotion experience. Previous studies has shown good reliability and validity of this measure, this questionnaire was not previously administered in ASD populations. Internal consistency in the current study was good (0.90 ≥ α ≥ 0.79).

#### Emotion Awareness

Children rated their awareness and understanding of their own emotions on two subscales of the Emotion Awareness Questionnaire (EAQ; Rieffe et al. 2008): differentiating emotions and bodily awareness of emotions. The subscale differentiating emotions contained 7 items and measured whether children were able to differentiate between their own emotions (e.g., “I am often confused or puzzled about what I am feeling [reversed-scored]”). Ratings were made on a 3-point scale ranging from 1 = (almost) not true to 3 = always true. A high score indicates good ability to differentiate between emotions. The subscale Bodily Awareness of Emotions measures whether children are aware of bodily changes related to emotions and consists of 5 items (e.g., “I don´t feel anything in my body when I am scared or nervous”). A high score indicated low bodily awareness, which was associated with more emotion awareness based on factor analysis (Rieffe et al. 2008). A total score of the 2 subscales were used as index for emotion awareness. The EAQ has shown to have good reliability and validity (Lahaye et al. 2011) and has been previously administered in children and adolescents with ASD (Rieffe et al. 2011). In the current study, internal consistency of this measure was acceptable (0.81 > α ≥ 0.69).

#### Worry/Rumination

The worry/rumination questionnaire for children (Jellesma et al. 2005; Miers et al. 2007) is a self-report measure, which assess the tendency of children to dwell on a problem instead of dealing with it in terms of solving or coping adaptively with the emotional impact of the situation. The questionnaire comprises 10 items and children are asked to rate the degree to which each item (e.g., When I have a problem, I think about it all the time) is true about them on a 3-point scale (1 = not true, 2 = sometimes true, 3 = often true). A high score indicates a high level of worry/rumination. This questionnaire has good reliability and validity and was previously administered in children and adolescents with ASD (Rieffe et al. 2011). In the current study, internal consistency was good (0.81 ≥ α ≥ 0.89).

### Outcome Measures

#### Disruptive Behavior Problems

The Child Symptom Inventory (CSI; Gadow and Sprafkin 1994; Dutch version by; Theunissen et al. 2012) is a behavior rating-scale to assess childhood disorders based on DSM-IV criteria. The parent-checklist was used to assess problems related to attention deficit hyperactivity disorder (ADHD), oppositional deviant disorder (ODD) and conduct disorder (CD). Seventeen items assessed the symptoms of ADHD (e.g., “Is quickly distracted”), eight items assessed symptoms of ODD (e.g., “Does things to deliberately annoy others”) and 15 items assessed symptoms of CD (e.g., “Has deliberately started fires”). Parents were asked to rate each symptom on a 4-point scale (1 = never and 4 = very often). A higher score indicated more disruptive behavior. Previous studies indicate that the CSI has satisfactory reliability and validity in community and ASD samples (Gadow and Sprafkin 2009). In the current study, internal consistency was high (0.93 > α ≥ 0.90).

For internalizing symptoms, three indices were taken: anxiety, depression, and somatic complaints.

#### Anxiety

The CSI was also used to assess problems related to generalized anxiety. Parents rated children’s *generalized anxiety symptoms* in the last six months on 7 items. Ratings were made on a 4-point scale ranging from 1 = never to 4 = very often. We used a total score of the 7 items. A higher score indicated more anxious feelings. Internal consistency was sufficient (0.82 > α ≥ 0.74).

#### Depression

Problems related to depression were measured with an adapted Dutch version of the Children’s Depression Inventory (CDI) (Kovacs 1985; Dutch version by; Timbremont et al. 2002). This self-report questionnaire includes 27 items that are related to specific depression symptoms (e.g., “*I am sad*”*)*. Ratings were on a three-point scale ranging from never/hardly true (1) to very true (3). The item pertaining to suicidal ideation was removed from the measure. In the analyses we used the total score of the 26 items. Higher scores on the CDI indicates higher depressive mood. CDI has good reliability and validity and has previously been administered in ASD populations (Lerner et al. 2012). Internal consistency in the current study was sufficient (0.86 > α ≥ 0.66).

*Somatic complaints* was measured by the Somatic Complaint List (SCL) (Jellesma et al. 2007). Children rated the frequency with which they experience certain somatic complaints such as a headache in the past four weeks on a 5-point scale (1 = never to 5 = very often). The scoring was reversed for the two positively formulated items. The SCL consists of 21 items; a high total score indicated more somatic complaints. Previous studies have shown that the SCL has good reliability and validity (Jellesma et al. 2007) and has previously been administered in ASD populations (Rieffe et al. 2011). Internal consistency in the current study was sufficient (0.83 > α ≥ 0.72).

### Procedure

Children were visited three times with approximately a 9-month time interval at home (*M*_T1 to 2_ = 8.77 months; *SD*_T1 to 2_ = 1.27; *M*_T2 to 3_ = 9.23, *SD*_T2 to 3_ = 1.17), at school or at their institution. As part of a larger study, children were asked to fill in several questionnaires on a laptop and to perform some experimental tasks. The test sessions took approximately 1 h each. It was emphasized that their responses would be anonymous. Parents were asked to complete questionnaires online or with paper and pencil. All participants were invited for the second and third wave. Nine participants (ASD: n6; TD: n = 5; attrition rate: 7.1%) in the second wave and 26 participants in the third wave (ASD: n = 9; TD: n = 17; attrition rate: 16.8%) indicated that they could not or did not want to participate anymore.

### Statistical Analyses

Statistical analyses were performed using the statistical software package for social sciences version 21.0 (SPSS Inc., Chicago). Sample characteristics were analyzed by independent *t* test. To test whether indices of emotion control at T1 predict problem behavior at T3, we used a hierarchical regression analyses for each problem behavior separately. In these analyses we entered as predictors diagnostic group (dummy coded: 1 = ASD, 0 = TD) in the first step, negative emotionality, emotion awareness and worry/rumination in the second step and the interaction between diagnostic group and the indices of emotion control in the third step. Outcome variables were indices of internalizing and externalizing behavior. All predictors were centered to the mean before entered in the regression analyses. Even though our predictors were moderately correlated with one another (see Supplementary Table 3), multicollinearity diagnostics indicated adequate tolerance levels. Note, controlling for IQ did not alter the results.

To analyze the developmental trajectory of behavior problems in boys with ASD compared to TD boys, we used a multilevel model approach (Singer and Willett 2003). One advantage of a multilevel approach is that it allows for hierarchy within data, such as observed in longitudinal data. In a longitudinal data set, time points are nested within participants and multilevel modeling can account for this data dependency. Another advantage of multilevel modeling is that it can handle missing data. In a multilevel model, cases with complete data at every time point are weighted more heavily. Importantly, as long as one time point of measurement is available, the case is included in the estimation of effects. In these analyses, time was treated as within-individual variable (*t*) (level 1) and group was included as between-individual variable (level 2). All mixed-models followed a formal model-fitting procedure. That is, we started with an unconditional means model that only included a fixed and random intercept, to allow for individual differences in starting points and account for the repeated nature of the data. The unconditional means model was compared to additional models that tested the grand mean trajectory of age [centered around 9 years (age of the youngest child)]. Thereafter we included diagnostic group and the interaction between age and diagnostic group, to examine whether the developmental trajectory of individuals with ASD differed from TD individuals. Preferred models had significantly lower Akaike Information Criterion values (AIC; Akaike 1974) and Bayesian Information Criterion (BIC; Schwarz 1978) values.

Finally, we tested whether *changes* in indices of emotion control could explain *change* in problem behavior (i.e., score of measurement 1–3 minus the score of measurement 1 for the indices of emotion control and problem behavior). We used a step-wise procedure including (1) change score of each predictor and (2) including the interactions with diagnostic group. In the results the best fitted models are described.

## Results

### Emotion Control Predicts Internalizing and Externalizing Behavior Problems in Boys

Table 2 depicts the results of the hierarchical regression analyses examining whether negative emotionality, emotion awareness and worry/rumination could predict problem behavior 18 months later. For disruptive behavior problems, diagnostic group and the interaction between group and worry/rumination contributed significantly to the prediction of externalizing behavior 18 months later (T3). For boys with ASD, high levels of worry/rumination were related to high levels of disruptive behavior at T3 (β = 0.95, *p* = .03), but not for TD boys (β = 0.28, *p* = .37).

**Table 2:** Results of hierarchical regression analyses examining effects of emotion dysregulation on T1 and problem behavior at T3

	Disruptive behavior		Anxiety		Depression		Somatic complaints	
	R^2^	β	R^2^	β	R^2^	β	R^2^	β
Step 1	0.39***		0.31***		0.03*		− 0.001	
Group		17.98***		4.56***		2.34*		
Step 2	0.43*		0.36*		0.15***		0.17***	
Group		15.21***		3.76***		1.18		− 0.35
Negative emotionality		0.79**		0.20*		0.25		0.21*
Poor emotion awareness		0.24		0.01		0.004		− 0.05
Worry/rumination		0.26		0.10		0.38**		0.27**
Step 3	0.46*		0.36		0.14		0.16	
Group		17.42***						
Negative emotionality (NE)		0.64						
Poor emotion awareness (PEA)		0.35						
Worry/rumination (W/R)		− 0.26						
Groups × NE		− 0.35						
Group × PEA		− 0.32						
Group × W/R		1.33*						

Unstandardized β coefficients and adjusted R^2^ are reported

*p < .05; **p < .01; ***p < .001

For symptoms of anxiety, only negative emotionality contributed significantly to the prediction of symptoms of anxiety at T3. That is, higher baseline levels of negative emotionality predicted more symptoms of anxiety at T3. For symptoms of depression, both diagnostic group and worry/rumination contributed to the prediction of symptoms of depression at T3. Boys with ASD showed more symptoms of depression at T3. Furthermore, boys—independent of diagnostic group—with higher levels of worry/rumination at T1 also showed more symptoms of depression at T3. For somatic complaints, diagnostic group was not a significant predictor. Baseline levels of negative emotionality and worry/rumination predicted somatic complaints at T3. Higher levels of negative emotionality and worry/rumination was predictive for higher levels of somatic complaints at T3.

### Developmental Trajectory of Internalizing and Externalizing Behavior in Boys with ASD

**Fig. 1:**
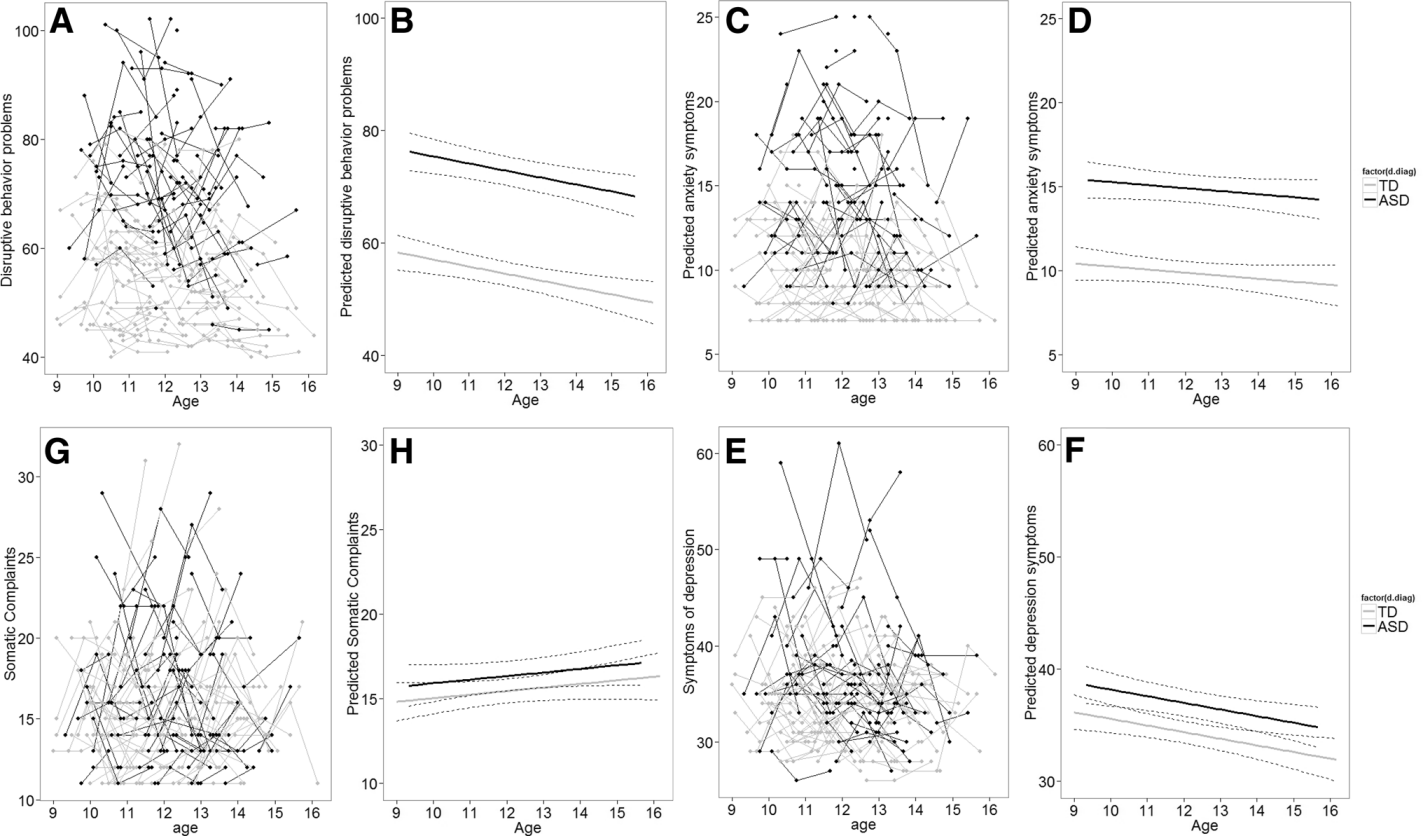
Longitudinal graphic representation of age at three time points and respectively internalizing and externalizing symptoms. **a, c, e, g** Participants are represented by individual lines. Participants measured only once are represented by points. **b, d, f, h** Predicted values for respectively internalizing, externalizing symptoms based on optimal fitting model

As can be seen in Fig. 1 and confirmed by the multilevel analyses, boys with ASD showed in general more disruptive behavior problems, symptoms of depression, anxiety, and somatic complaints compared to TD boys (see Supplementary Table 2). There was a negative linear trend for reported disruptive behavior problems (b = − 1.03, *t* = 2.18, *p* = .03), indicating a decrease in disruptive behavior over time. For somatic complaints, there was a positive linear trend (b = 0.37, *t* = 2.10, *p* = .04), indicating an increase in reported somatic complaints over time. The developmental trajectory of symptoms of anxiety and depression was rather stable. So, we observed no significant change in symptoms of anxiety and depression over time. For all outcome measures there were no differences between groups in developmental trajectory.

### Emotion Control Predicts the Developmental Trajectory of Internalizing and Externalizing Problems

**Fig. 2:**
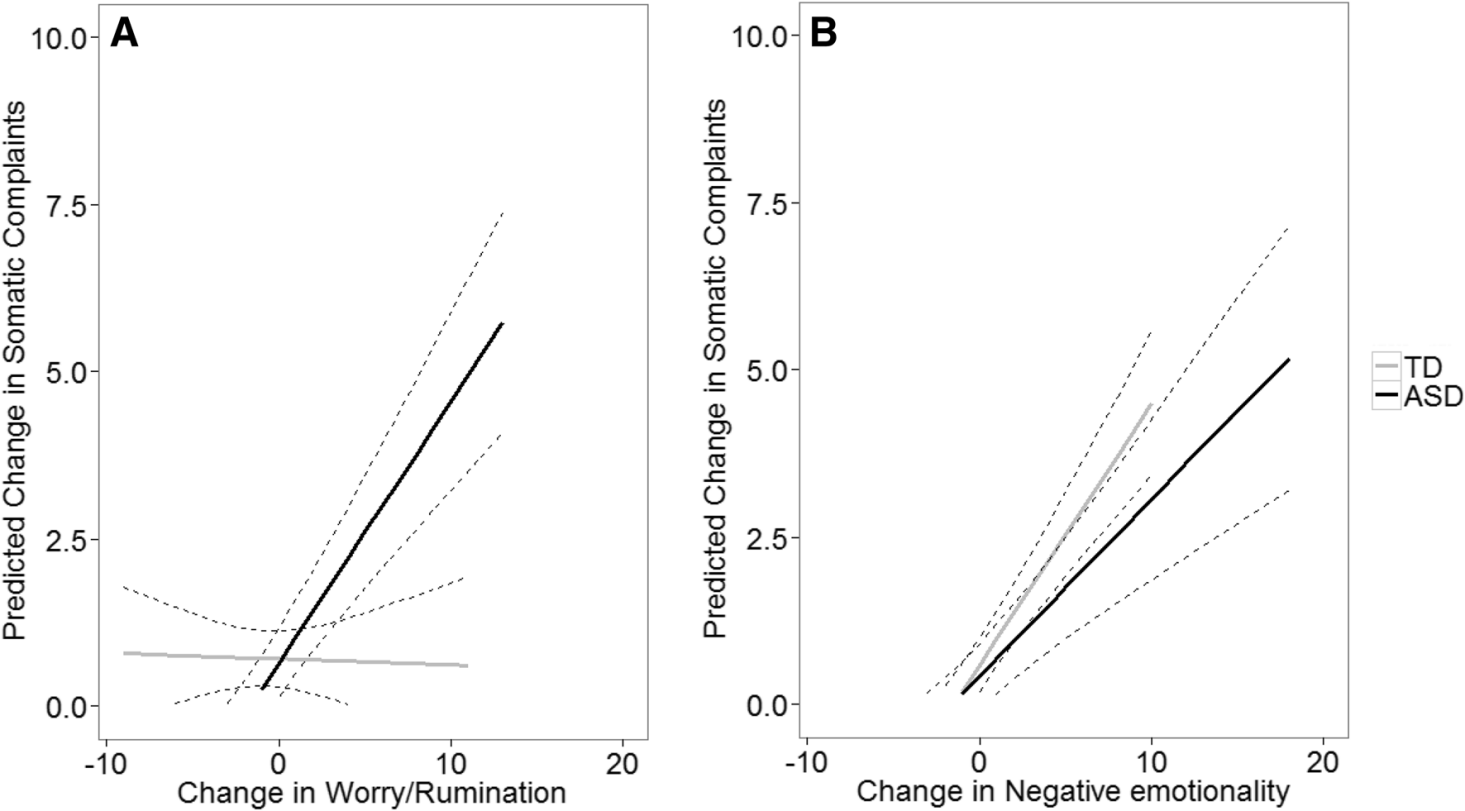
Longitudinal graphic representation of the interaction between emotion control and group on somatic complaints. **a** Change in worry/rumination over time predicts the developmental trajectory of somatic complaints in children and adolescents with ASD. **b** Change in negative emotionality over time predicts the trajectory of somatic complaints in both groups, but stronger for children and adolescents with a TD development. The graphs represent the single relation between one emotion control index and somatic complaints, without controlling for other variables that were included in the mixed model

Table 3 depicts the results of the multilevel models for the longitudinal relation between the three indices of emotion control and internalizing and externalizing behavior problems. The multilevel models without interaction terms fitted best for disruptive behavior problems, symptoms of anxiety, and symptoms of depression. For disruptive behavior problems, an increase in worry/rumination predicted increase in disruptive behavior problems. Also for symptoms of anxiety was worry/rumination a significant predictor. An increase in worry/rumination was related to an increase in anxiety symptoms. For symptoms of depression, both change in worry/rumination and change in negative emotionality predicted change in symptoms of depression. Interestingly, for somatic complaints the multilevel model including interaction terms with group fitted best. Change in worry/rumination, negative emotionality, and emotion awareness predicted change in somatic complaints. Furthermore, there was also a significant interaction effect between diagnostic group and worry/rumination and diagnostic group and negative emotionality. As can be seen in Fig. 2a, increase in worry/rumination related to increase in somatic complaints specifically in the ASD group. In Fig. 2b, increase in negative emotionality related to increase in somatic complaints in both groups, but this relation was stronger in the TD group than in the ASD group.

**Table 3:** Results of the linear mixed model analyzing the effect of *change* in emotion dysregulation affects *change* in problem behavior

	Δ Disruptive behavior	Δ Anxiety	Δ Depression	Δ Somatic complaints
Fixed effects				
Intercept	− 0.21	0.04	− 0.30	0.43*
Group	− 0.46	− 0.01	0.29	0.27
Δ Negative emotionality	− 0.01	− 0.03	0.28***	0.42***
Δ Emotion awareness	− 0.03	0.05	− 0.06	− 0.12*
Δ Worry/rumination	0.24*	0.07^#^	0.14*	− 0.15**
Group × Δ negative emotionality				− 0.23**
Group × Δ emotion awareness				0.08
Group × Δ worry/rumination				0.41***
Random effects				
ID	10.33***	0.56*	5.01***	1.31***
AIC	2408.08	1566.18	2352.02	1916.77
BIC	2415.88	1573.98	2360.05	1924.79
Degrees of freedom	7	7	7	10

^#^*p* < .08; *p < .05; **p < .01; ***p < .001

## Discussion

Many children and adolescents with ASD show emotional and behavior problems, in addition to their core symptoms (Gadow et al. 2012; Simonoff et al. 2008). It is therefore important to investigate possible underlying mechanisms to explain this co-occurrence of symptomatology and to provide tools for prevention and intervention of these problems in children and adolescents with ASD. In the current longitudinal study we examined the predictive role of three indices of emotion control that are assumed to play a key role in the development of additional problem behavior in children and adolescents with ASD. Our main findings are: (1) baseline level of worry/rumination was a risk factor for the development of externalizing behavior symptoms 18 months later, yet only for boys with ASD, (2) the developmental trajectory of internalizing and externalizing behavior symptoms did not differ between boys with and without ASD, (3) increase in worry/rumination over time was related to the development of more externalizing behavior problems in boys with and without ASD and (4) increase in worry/rumination and increase in negative emotionality contributed both to the development of more internalizing behavior symptoms in boys with and without ASD, yet the longitudinal relation between worry/rumination and somatic complaints was specific for boys with ASD. Below we will discuss the theoretical and clinical implications of our findings.

### Developmental Trajectory of Internalizing and Externalizing Behavior in Both Groups

Consistent with previous studies, we found that boys with ASD showed more internalizing and externalizing behavior problems than boys without ASD (Simonoff et al. 2012, 2008). However, the pace with which these symptoms develop over time did not differ between groups. Moreover, we did not find an increase in symptoms of depression and generalized anxiety. This finding seems to be in contrast with previous studies showing that the level of internalizing problems increases during adolescence (e.g., Paus et al. 2008) and are more pronounced in adolescents with ASD (Gotham et al. 2015; Kuusikko et al. 2008). However, other longitudinal studies reported no age effects or even a decrease in internalizing symptoms in adolescent and adults with ASD (Andersen et al. 2015; Shattuck et al. 2007; Woodman et al. 2015). The mixed findings in the literature, may be explained by differences in sample characteristics like gender distribution, level of IQ, age range, and including individuals with or without comorbid diagnoses. Indeed, a previous study found that the increase in internalizing symptoms in adolescents with ASD was driven by girls, whereas boys with ASD did not show age effects on reported internalizing symptoms (Gotham et al. 2015). Moreover, in the current study we used a homogenous sample of high-functioning boys with ASD without a comorbid diagnosis, whereas previous studies that found an increase in internalizing problems used heterogeneous samples of individuals with ASD (Gotham et al. 2015; Kuusikko et al. 2008).

Furthermore, the current findings revealed a decrease in externalizing behavior with age for boys with and without ASD. This finding is in line with cross-sectional studies reporting a negative relation between aggressive behavior and age (Farmer et al. 2015; Kanne and Mazurek 2011), yet others report a relatively stable relation of externalizing behavior over time (Bader and Barry 2014). The number of longitudinal studies are, however, limited and essential to assess the developmental trajectories of problem behavior.

Adolescence is a time period characterized by strong changes in behavior and biology (Steinberg and Morris 2001). It is also a developmental period sensitive to the emergence of psychiatric problems (Paus et al. 2008). Importantly, the current findings indicate that this period is not an additional risk factor for boys with ASD to develop comorbid symptomatology. Moreover, it seems that their heightened sensitivity to emotional and behavior problems that are beyond their core diagnostic symptoms already exist during (early) childhood. Given their social and communication difficulties, children with ASD are less able to participate in family and social life. Speculatively, this diminished access to a social learning environment might affect their opportunity to practice and achieve emotion control directly and might explain why comorbid symptomatology emerges prior to the teenage years.

### Worry/Rumination as a Risk Factor for Developing Externalizing Symptomatology

In line with previous findings we found that boys with ASD show more disruptive and aggressive behavior than boys without ASD (e.g., Farmer and Aman 2011). As has been noted in previous studies, boys with ASD might act out their frustration and negative thoughts (Patel et al. 2017). Indeed, in the current study we demonstrate that baseline levels of worry/rumination was a risk factor for disruptive behavior problems 18 months later, yet only for boys with ASD. This indicates that dealing with daily problems by repetitive negative thinking has a stronger impact for boys with ASD than for boys without ASD. Possibly, boys with ASD worry/ruminate about other daily problems than boys without ASD. It is often assumed that high-functioning people with ASD are well aware of their social problems and appear to wish this could be different (Attwood 2000). This awareness of social disconnectedness might be an important source for daily problems and worries, specifically during adolescence when peers play an increasingly important role in daily life. A recent cross-sectional study indeed demonstrated that adolescents with ASD reported more anger rumination than TD youth (Patel et al. 2017). It bears mentioning that in the current study boys with ASD did not worry/ruminate more than boys without ASD (see Supplementary Table 1).

Furthermore, we showed that an increase in worry/rumination over time also contributed to the prediction of more externalizing behavior problems over time for boys with and without ASD. This finding is in line with a recent large-scale longitudinal study showing that worry/rumination in TD boys is a risk factor for later aggressive behavior (McLaughlin et al. 2014) and a cross-sectional study in adolescents with ASD that showed that *anger* rumination was associated with aggressive behavior (Patel et al. 2017). As will be discussed in more detail later, this finding emphasizes the important role of worry/rumination as an underlying mechanism explaining the development of multiple psychopathological symptoms.

### Emotion Control as a Risk Factor for Developing Internalizing Symptomatology

Boys with ASD showed a heightened sensitivity to develop internalizing behavior symptoms. In the current study, however, we merely identified general risk factors for the development and maintenance of internalizing problems for boys with and without ASD.

Our findings showed that dealing with daily problems by repeatedly and negatively thinking about these problems increases the risk for developing internalizing behavior problems 18 months later (i.e., symptoms of depression, somatic complaints) in boys with and without ASD. Furthermore, increase in frequency of worry/rumination also contributed to the prediction of increase in internalizing symptoms (depression, anxiety, somatic complaints). These findings combined with the observed longitudinal relation between worry/rumination and externalizing behavior symptoms confirm that worry rumination is a transdiagnostic factor underlying multiple types of psychopathology (Aldao et al. 2010; Ehring and Watkins 2008; Nolen-Hoeksema 2000) that are beyond the core symptoms of ASD.

It should be noted that the longitudinal relation between worry/rumination and somatic complaints was most apparent in the ASD group. A previous study demonstrated a cross-sectional relation between anger related rumination and somatic complaints in TD children (Miers et al. 2007). Possibly, worry/rumination is a stronger stressor for boys with ASD than for boys without ASD. It has been argued that preservative cognition, like worry/rumination, moderate the relation between stressors and somatic complaints by prolonging the stress-level (Brosschot et al. 2006). So, repetitive negative thinking in response to daily issues might induce and prolong the stress responses related to these daily hazards more in boys with ASD, which in turn affect their physiological well-being stronger. This hypothesis should be tested in future studies.

Another risk factor for internalizing symptomatology is a regular experience of negative emotions (Gross and John 2003; McLaughlin et al. 2011; Moses and Barlow 2006). In line with this, we showed that for boys with and without ASD baseline levels of negative emotionality was a predictor for internalizing symptoms (i.e., anxiety, somatic complaints) 18 months later. Furthermore, we showed that an increase in negative emotionality over time was related to an increase in internalizing behavior problems (i.e., depression, somatic complaints) over time. Note that this longitudinal relation between negative emotionality and somatic complaints was evident for all boys, albeit stronger for TD boys. The frequent experience of anger, fear and/or sadness reveales a child’s inability to effectively deal with daily emotional experiences. Maintaining a negative mood state affects the way a child approach new situations. For example, negative mood negatively affects basic cognitive processes, like memory, attention and interpretation (e.g., Gendolla 2000), which in turn, are important in regulating emotions (Gross 2013; Sheppes et al. 2015). In this way, a vicious circle may emerge resulting in an increased risk to delevop and maintain internalizing symptomatology.

Remarkably, the ability to differentiate between emotional states and be aware of bodily changes that are accompanied by those states did not uniquely contribute to the prediction of internalizing and externalizing behavior problems, except for the development of somatic complaints. This finding is unexpected, given that understanding of one’s own emotions is critical for the experience and regulation of emotions (Barrett et al. 2001; Lambie and Marcel 2002; Rieffe et al. 2008). Moreover, alexithymia—a broader construct that also involves emotion awareness—is associated with internalizing problems in youth with ASD (Milosavljevic et al. 2016). This discrepancy in results may be explained by differences in research design. Note, that alexithymia is often examined in isolation, whereas we investigated three indices of emotion control. As can be seen in Supplementary Table 3, we found correlations between emotion awareness at T1 and depression and somatic complaints at T3.

### Limitations

The current study had several strengths including a longitudinal design, an adequate sample size, and a typical developing control group. However, the following limitations should be considered when interpreting the current findings. First, our sample included high functioning boys with ASD without co-occurring psychiatric problems. Hence, the boys with ASD in our sample may not reflect a representative sample of boys with ASD; showing less severe problem behavior. Yet, our selection criteria ascertains group comparisons and thereby that the findings in this study can be attributed to the diagnosis of ASD. Second, our results on the developmental pattern of internalizing and externalizing behavior problems in boys with ASD should be interpreted with caution, given that in the study period participants with ASD and their parents received psychoeducation on ASD. Hence, this may have positively affected their developmental trajectories of internalizing and externalizing behavior, showing less severe problem behavior. Third, we focused on only three processes related to emotion control. Given the complex nature of emotion control and its relation to emotional and behavioral problems, we could only clarify a small part of this relationship. For example, we only selected worry/rumination as a measure of cognitive emotion regulation, but other maladaptive strategies for example catastrophizing and self-blame may also be related to the development of psychopathology in children and adolescents with ASD (Mazefsky and White 2014; Rieffe et al. 2014). It would be interesting to incorporate both adaptive and maladaptive cognitive emotion regulation strategies to gain a more detailed understanding of possible risk as well as protective factors for the development of problem behavior. Related to this issue, we used one measure for each construct of emotion regulation. Given the complexity of these measures, it is highly likely that they are contaminated by random- and systematic-error. It is well established in the literature that a single indicator of any given construct can rarely, if ever, be viewed as a pure measure of the construct (Kline 1998). Consequently, future studies using multiple measures of emotion control incorporating perhaps latent variable approaches extracting common variance to these concepts may provide a clearer picture of the relations between different aspects of emotion control and psychopathology in ASD. Moreover, one cannot expect a single emotion control profile to characterize all children with ASD. Rather, just as there are individual differences in the core ASD symptom manifestation there ought to be individual differences or variability in the various processes related to emotion control. It is therefore important for future research to determine emotion control profiles in children with ASD on an individualized basis that are (mal)adaptive for their development. Fourth, in the current study we assessed emotion control with self-report questionnaires and subsequently rely on the ability of our participants to describe their emotions. Even though growing body of research suggests that introspection does not form a problem for adolescents with ASD (Bird and Cook 2013; Milosavljevic et al. 2016; Rieffe et al. 2011), the quality of this introspection might differ between adolescents with ASD and their TD controls. Moreover, we used self-report measures to index emotion control, depression, and somatic complaints and parent-reports to measure generalized anxiety and disruptive behavior problems. The use of different informants for the outcome measures may have affected the current findings. Previous studies have shown that informant agreement is only modest (Rescorla et al. 2013), but that informant-agreement does not differ for children and adolescents with ASD compared to their TD peers (Stratis and Lecavalier 2015). Possibly, modest informant agreement has lowered our power to detect associations between self-reported emotion control with parent-reported symptoms of generalized anxiety and disruptive behavior problems compared to detecting associations between self-reported emotion control with self-reported symptoms of depression and somatic complaints. This might be specifically the case for the relation between emotion control and generalized anxiety, given that it is difficult for parents to observe and report internalizing behavior during adolescence when children become more and more independent from their parents.

## Conclusion

The current longitudinal study provided important insights on the role of emotion control in developing and maintaining internalizing and externalizing behavior problems in boys with and without ASD. We showed that worry/rumination is an important risk factor for developing externalizing behavior problems in boys with ASD 18 months later. Moreover, increase in the frequency of this negative thinking style also contributed to the prediction of increase in internalizing and externalizing behavior problems over time in boys with and without ASD. This implies that worry/rumination is a key factor underlying the development of a broad spectrum of psychiatric symptomatology. For clinical practice, a focus on transdiagnostic factors, such as worry/rumination may prove useful to include in prevention and treatment programs for children and adolescents with ASD aimed at preventing of the development of comorbidity (Weiss 2014).

## Electronic supplementary material

Below is the link to the electronic supplementary material.

Supplementary material 1 (DOCX 34 KB)
