# Supplementary material for: Emotion Control Predicts Internalizing and Externalizing Behavior Problems in Boys With and Without an Autism Spectrum Disorder
Source: J Autism Dev Disord. 2018 Mar 6;48(8):2727–39. doi: 10.1007/s10803-018-3519-8 (PMC6061024; doi:10.1007/s10803-018-3519-8)
Supplement: Supplementary file 1 — Supplementary material 1 (DOCX 34 KB) [file 10803_2018_3519_MOESM1_ESM.docx]

**Supplementary Table 1**. Mean and standard deviations of all predictors and outcome variables for each group separately.

|  |  | **ASD** | |  | **Controls** | |
| --- | --- | --- | --- | --- | --- | --- |
|  | n | M | *SD* | n | M | *SD* |
| **T1** |  |  |  |  |  |  |
| Disruptive behavior^***^ | 60 | 73.40 | *13.16* | 86 | 54.91 | *10.11* |
| Anxiety^***^ | 60 | 14.83 | *4.30* | 86 | 9.90 | *2.66* |
| Depression^**^ | 66 | 37.09 | *5.88* | 89 | 34.51 | *4.43* |
| Somatization^*^ | 65 | 16.05 | *3.88* | 89 | 14.80 | *3.00* |
| Negative Emotionality^***^ | 65 | 18.40 | *4.70* | 89 | 15.92 | *3.34* |
| Poor Emotion Awareness | 64 | 26.09 | *4.93* | 89 | 26.54 | *4.30* |
| Worry/Rumination | 65 | 19.63 | *4.54* | 89 | 18.29 | *4.24* |
| **T2** |  |  |  |  |  |  |
| Disruptive behavior^***^ | 56 | 70.55 | *11.61* | 77 | 54.66 | *9.43* |
| Anxiety^***^ | 56 | 15.20 | *4.61* | 77 | 10.01 | *2.40* |
| Depression^**^ | 61 | 37.65 | *6.26* | 84 | 34.60 | *4.54* |
| Somatization | 59 | 16.75 | *4.25* | 84 | 16.00 | *3.98* |
| Negative Emotionality | 59 | 17.32 | *5.44* | 84 | 16.68 | *4.05* |
| Poor Emotion Awareness | 60 | 27.38 | *4.95* | 83 | 26.60 | *3.91* |
| Worry/Rumination | 59 | 17.95 | *5.14* | 84 | 17.36 | *4.22* |
| **T3** |  |  |  |  |  |  |
| Disruptive behavior^***^ | 50 | 70.97 | *12.81* | 65 | 53.47 | *9.67* |
| Anxiety^***^ | 56 | 14.32 | *3.74* | 65 | 9.77 | *2.95* |
| Depression^*^ | 57 | 35.60 | *6.86* | 71 | 33.45 | *4.67* |
| Somatization | 56 | 16.43 | *4.16* | 71 | 15.79 | *3.71* |
| Negative Emotionality | 56 | 17.64 | *5.58* | 71 | 16.45 | *4.20* |
| Poor Emotion Awareness | 56 | 27.41 | *4.21* | 71 | 26.23 | *4.27* |
| Worry/Rumination | 56 | 17.36 | *4.57* | 71 | 17.17 | *3.69* |

^*^ *p* < .05, ^**^ *p* < .01, ^***^ *p* <.001

**Supplementary Table 2**. Results of the linear mixed model to assess the developmental trajectory of each outcome variable. Estimates of the beta’s are reported.

|  | Disruptive behavior | Symptoms of anxiety | Symptoms of depression | Somatic Complaints |
| --- | --- | --- | --- | --- |
| **Model 1** |  |  |  |  |
| Intercept | 61.84^***^ | 11.98^***^ | 35.30^***^ | 15.86^***^ |
| AIC/BIC | 2884.46/  2892.41 | 1983.86/  1991.81 | 2571.19/  2579.30 | 2215.20/  2223.29 |
| Df | 3 | 3 | 3 | 3 |
|  |  |  |  |  |
| **Model 2** |  |  |  |  |
| Intercept | 64.94^***^ | 12.27^***^ | 36.96^***^ | 15.15^***^ |
| Age | -0.97^***^ | -0.09 | -0.52^**^ | 0.22 |
| AIC/BIC | 2860.64/  2872.57 | 1976.65/  1984.59 | 2565.94/  2574.05 | 2214.80/  2222.89 |
| Df | 4 | 4 | 4 | 4 |
|  |  |  |  |  |
| **Model 3** |  |  |  |  |
| Intercept | 57.66^***^ | 10.41^***^ | 35.45^***^ | 14.34^***^ |
| Age | -1.03^*^ | -0.16 | -0.40 | 0.37^*^ |
| Group | 19.61^***^ | 4.98^***^ | 3.93^*^ | 2.15^*^ |
| Age x Group | -0.61 | -0.01 | -0.40 | -0.41 |
| AIC/BIC | 2773.26/  2781.18 | 1904.10/  1912.02 | 2551.08/  2559.18 | 2210.53/  2218.61 |
| df | 6 | 6 | 6 | 6 |

^*^ *p* < .05, ^**^ *p* < .01, ^***^ *p* <.001

**Supplementary Table 3.** Correlation table of all outcome and predictor variables at all Timepoints.

|  | 1 | 2 | 3 | 4 | 5 | 6 | 7 | 8 | 9 | 10 | 11 | 12 | 13 | 14 | 15 | 16 | 17 | 18 | 19 | 20 | 21 |
| --- | --- | --- | --- | --- | --- | --- | --- | --- | --- | --- | --- | --- | --- | --- | --- | --- | --- | --- | --- | --- | --- |
| 1. Disruptive behavior T1 | 1 |  |  |  |  |  |  |  |  |  |  |  |  |  |  |  |  |  |  |  |  |
| 1. Disruptive behavior T2 | .83^***^ | 1 |  |  |  |  |  |  |  |  |  |  |  |  |  |  |  |  |  |  |  |
| 1. Disruptive behavior T3 | .82^***^ | .88^***^ | 1 |  |  |  |  |  |  |  |  |  |  |  |  |  |  |  |  |  |  |
| 1. Generalized Anxiety T1 | .79^***^ | .66^***^ | .59^***^ | 1 |  |  |  |  |  |  |  |  |  |  |  |  |  |  |  |  |  |
| 1. Generalized Anxiety T2 | .69^***^ | .75^***^ | .66^***^ | .85^***^ | 1 |  |  |  |  |  |  |  |  |  |  |  |  |  |  |  |  |
| 1. Generalized Anxiety T3 | .69^***^ | .62^***^ | .71^***^ | .77^***^ | .77^***^ | 1 |  |  |  |  |  |  |  |  |  |  |  |  |  |  |  |
| 1. Depression T1 | .42^***^ | .42^***^ | .47^***^ | .36^***^ | .33^***^ | .44^***^ | 1 |  |  |  |  |  |  |  |  |  |  |  |  |  |  |
| 1. Depression T2 | .41^***^ | .51^***^ | .45^***^ | .33^***^ | .40^***^ | .42^***^ | .57^***^ | 1 |  |  |  |  |  |  |  |  |  |  |  |  |  |
| 1. Depression T3 | .46^***^ | .48^***^ | .48^***^ | .36^***^ | .41^***^ | .39^***^ | .44^***^ | .66^***^ | 1 |  |  |  |  |  |  |  |  |  |  |  |  |
| 1. Somatic Complaints T1 | .31^***^ | .25^**^ | .36^***^ | .27^***^ | .28^***^ | .39^***^ | .56^***^ | .41^***^ | .35^***^ | 1 |  |  |  |  |  |  |  |  |  |  |  |
| 1. Somatic Complaints T2 | .16 | .21^*^ | .21^*^ | .22^*^ | .26^**^ | .37^***^ | .40^***^ | .51^***^ | .45^***^ | .60^***^ | 1 |  |  |  |  |  |  |  |  |  |  |
| 1. Somatic Complaints T3 | .22^*^ | .26^**^ | .25^**^ | .28^**^ | .39^***^ | .41^***^ | .25^**^ | .44^***^ | .56^***^ | .49^***^ | .69^***^ | 1 |  |  |  |  |  |  |  |  |  |
| 1. Negative Emotionality T1 | .32^***^ | .37^***^ | .45^***^ | .27^***^ | .31^***^ | .41^***^ | .52^***^ | .51^***^ | .33^***^ | .54^***^ | .47^***^ | .33 | 1 |  |  |  |  |  |  |  |  |
| 1. Negative Emotionality T2 | .16 | .27^**^ | .23^*^ | .19^*^ | .22^***^ | .25^**^ | .36^**^ | .47^**^ | .34^**^ | .30^**^ | .58^***^ | .37^***^ | .48^***^ | 1 |  |  |  |  |  |  |  |
| 1. Negative Emotionality T3 | .24^**^ | .33^***^ | .29^**^ | .29^**^ | .38^***^ | .38^***^ | .25^*^ | .39^***^ | .53^***^ | .33^***^ | .51^***^ | .65^***^ | .39^***^ | .61^***^ | 1 |  |  |  |  |  |  |
| 1. Emotion awareness T1 | -.09 | -.09 | -.10 | -.05 | -.15 | -.17 | -.24^**^ | -.23^**^ | -.20^*^ | -.40^***^ | -.36^***^ | -.27^**^ | -.32^***^ | -.21^*^ | -.27^***^ | 1 |  |  |  |  |  |
| 1. Emotion awareness T2 | -.02 | -.07 | .03 | -.08 | -.23^**^ | -.09 | -.07 | -.26^**^ | -.25^**^ | -.15 | -.39^***^ | -.33^***^ | -.11 | -.47^***^ | -.46^***^ | .37^***^ | 1 |  |  |  |  |
| 1. Emotion awareness T3 | -.01 | -.04 | -.05 | -.05 | -.12 | -.14 | -.10 | -.25^**^ | -.29^**^ | -.18^*^ | -.42^***^ | -.47^***^ | -.18^*^ | -.33^***^ | -.48^***^ | .48^***^ | .57^***^ | 1 |  |  |  |
| 1. Worry/Rumination T2 | .14 | .12 | .24^*^ | .18^*^ | .16 | .27^**^ | .36^***^ | .27^***^ | .37^***^ | .41^***^ | .47^***^ | .40^***^ | .44^***^ | .33^***^ | .37^***^ | -.52^***^ | -.28^***^ | -.39^***^ | 1 |  |  |
| 1. Worry/Rumination T3 | .18^*^ | .26^***^ | .19^*^ | .19^*^ | .21^*^ | .19^*^ | .21^*^ | .39^***^ | .40^***^ | .23^**^ | .46^***^ | .38^***^ | .32^***^ | .58^***^ | .53^***^ | -.32^***^ | -.61^***^ | -.40^***^ | .52^***^ | 1 |  |
| 1. Worry/Rumination T1 | .12 | .23^*^ | .23^*^ | .16 | .26^**^ | .30^**^ | .17 | .31^***^ | .38^***^ | .16 | .43^***^ | .52^***^ | .18^*^ | .43^***^ | .59^***^ | -.26^*^ | -.46^***^ | -.57^***^ | .44^***^ | .65^***^ | 1 |

* p < .05; ** p < .01; *** p <.001

**Supplementary Table 4.** ICC for all outcome and predictor variables between Timepoint 1, 2, and 3

|  | ICC single measure | ICC average measure |
| --- | --- | --- |
| Disruptive behavior | .85 | .95 |
| Anxiety | .81 | .93 |
| Depression | .55 | .79 |
| Somatic complaints | .58 | .80 |
|  |  |  |
| Negative emotionality | .48 | .73 |
| Emotion Awareness | .47 | .73 |
| Worry/Rumination | .52 | .76 |
